# The 1-aminocyclopropane-1-carboxylic acid deaminase-producing *Streptomyces violaceoruber* UAE1 can provide protection from sudden decline syndrome on date palm

**DOI:** 10.3389/fpls.2022.904166

**Published:** 2022-07-27

**Authors:** Khawla J. Alwahshi, Gouthaman P. Purayil, Esam Eldin Saeed, Haneen A. Abufarajallah, Shama J. Aldhaheri, Synan F. AbuQamar, Khaled A. El-Tarabily

**Affiliations:** ^1^Department of Biology, United Arab Emirates University, Al-Ain, United Arab Emirates; ^2^Research Station Section, Abu Dhabi Agriculture and Food Safety Authority, Abu Dhabi, United Arab Emirates; ^3^Khalifa Center for Genetic Engineering and Biotechnology, United Arab Emirates University, Al-Ain, United Arab Emirates; ^4^Harry Butler Institute, Murdoch University, Murdoch, WA, Australia

**Keywords:** actinobacteria, biocontrol, date palm, *Fusarium solani*, rhizosphere, plant–microbe interaction, sudden decline syndrome

## Abstract

In the United Arab Emirates (UAE), sudden decline syndrome (SDS) is one of the major fungal diseases caused by *Fusarium solani* affecting date palm plantations. To minimize the impact of the causal agent of SDS on date palm, native actinobacterial strains isolated from rhizosphere soils of healthy date palm plants were characterized according to their antifungal activities against *F. solani* DSM 106836 (*Fs*). Based on their *in vitro* abilities, two promising biocontrol agents (BCAs), namely *Streptomyces tendae* UAE1 (*St*) and*Streptomyces violaceoruber* UAE1 (*Sv*), were selected for the production of antifungal compounds and cell wall degrading enzymes (CWDEs), albeit their variations in synthesizing 1-aminocyclopropane-1-carboxylic acid (ACC) deaminase (ACCD). Although both isolates showed antagonism when applied 7 days before the pathogen in the greenhouse experiments, the ACCD-producing *Sv* was relatively superior in its efficacy against SDS over the non-ACCD-producing *St*. This was evident from the symptoms of SDS in diseased date palm seedlings which were greatly reduced by *Sv* compared to *St*. On a scale of 5.0, the estimated disease severity indices in *Fs*-diseased seedlings were significantly (*P* < 0.05) reduced from 4.8 to 1.5 and 0.5 by *St* and *Sv*, respectively. Thus, the number of conidia of *Fs* recovered from plants pre-treated with both BCAs was comparable, but significantly (*P* < 0.05) reduced compared to plants without any BCA treatment. In addition, a significant (*P* < 0.05) decrease in ACC levels of both the root and shoot tissues was detected in*Sv* + *Fs* seedlings to almost similar levels of healthy seedlings. However, *in planta* ACC levels highly increased in seedlings grown in soils infested with the pathogen alone or amended with *St* prior to *F. solani* infestation (*St* + *Fs*). This suggests a major role of ACCD production in relieving the stress of date palm seedlings infected with *F. solani*, thus supporting the integrated preventive disease management programs against this pathogen. This is the first report of effective rhizosphere actinobacterial BCAs to provide protection against SDS on date palm, and to help increase agricultural productivity in a more sustainable manner in the UAE and the other arid regions.

## Introduction

Date palm (*Phoenix dactylifera* L.) is an important traditional tree cultivated in the arid region. Like other countries in the Arabian Gulf, the United Arab Emirates (UAE) consider dates as a significant source of food production that play a vital role in the food security of the region (Mahmoudi et al., 2008). Due to its existence in such harsh and competitive environments, date palm constantly interacts with pathogenic microorganisms present in its niche. Several fungi have been recorded as causal pathogens of diseases on date palms. The fungal pathogens, *Thielaviopsis punctulata*, *Thielaviopsis paradoxa*, *Omphalia pigmentata*, *Omphalia tralucida*, *Mycosphaerella tassiana*, and *Graphiola phoenicis* can cause foliar diseases, root diseases, and false smut on date palm in Oman, Qatar, Saudi Arabia, and the UAE (Al-Sadi et al., 2012; Al-Naemi et al., 2014; Saeed et al., 2016; Alhudaib et al., 2022). Sudden decline syndrome (SDS), also known as *Fusarium* wilt disease, of date palm is also caused by different fungal species that belong to *Fusarium* (Al-Hammadi et al., 2019). Bayoud disease, caused by the soil-borne fungal pathogen, *Fusarium oxysporum* f. sp. *albedinis*, is undoubtedly the most destructive disease affecting date palm in North Africa (Sedra, 2013). *Fusarium solani* has previously been identified as the causal agent of SDS on date palms in the UAE (Alwahshi et al., 2019).

In general, SDS causes severe damages in date palm plants that live in warm and dry regions (Shabani and Kumar, 2013). Plants growing in cool and wet areas may also show symptoms but at slower rates. *F. solani* is a soil-borne vascular fungal pathogen that attacks plants through the roots (Mansoori and Kord, 2006). The life cycle of this species can be divided into dormant, parasitic, and saprophytic phases (Okungbowa and Shittu, 2012). In the dormant phase, the germination of the fungal resting structures present in the soil is inhibited. Thus, this can be overcome by the released carbon and nitrogen (N) from root exudates in the rhizosphere of host plants. *Fusarium* spp. enter the parasitic stage by penetrating the roots through the root tip or at the sites of lateral root formation. This fungal pathogen can colonize the vascular (xylem) tissues after crossing the endodermis, spreads to the neighboring xylem elements, and sporulates until it colonizes the xylem tissues of the whole plant; thus, resulting in the accumulation of fungal biomass (Mansoori and Kord, 2006). At the saprophytic stage, *F. solani* can produce its conidia on infected plant tissues that may be dispersed and start another cycle of disease (Shabani and Kumar, 2013). In general, this fungus can persist in soil for several years without a host. The opportunistic fungus can attack old, weak, or injured plants, with similar symptoms of SDS that can be recognized on most date palm cultivars (Bokhary, 2010). Disease symptoms on date palm can be first observed on the lowest outer leaves of the middle crown, followed by yellowing at the base of the leaves/fronds, and move upward (Maitlo et al., 2016). As the disease progresses, leaves gradually turn white on one side before developing on the other side. Finally, the diseased plants are crinkled, leaves are dried, and eventually die from few days to several weeks after infection.

Today, farmers are primarily dependent on the application of chemical fungicides to inhibit the growth or prevent the spread of fungal pathogens and their spores, which in turn, protects crop plants (Youssef et al., 2019). In the UAE, Score ^®^ (difenoconazole) and Cidely ^®^ Top (difenoconazole and cyflufenamid) are effective chemical treatments for black scorch disease and SDS on date palm (Saeed et al., 2016, 2017b; Alwahshi et al., 2019), dieback on mango (Kamil et al., 2018), and stem canker on royal poinciana (Al Raish et al., 2020). However, the extensive use of chemicals has resulted in the emergence of fungicide-resistant pathogens and other concerns of the residual effects on the environment and human health. Biocontrol agents (BCAs) against phytopathogenic fungi (Al Hamad et al., 2021; Al Raish et al., 2021), botanical and microbial fungicides (Yoon et al., 2013; Moreno-Gavíra et al., 2021), agronanotechnology (Atiq et al., 2020), and deactivation and evacuation of fungal cells (El-Baky and Amara, 2021) can be employed as “green” strategies to control plant fungal diseases. Integrated disease management (IDM) combining BCAs with fungicides can be another option to reduce the impact of fungicides as well as to effectively manage plant diseases (Saeed et al., 2017b; Kamil et al., 2018; Zapata-Sarmiento et al., 2020). In that sense, actinobacteria have been constantly proposed as possible candidates to replace chemical fungicides (Saeed et al., 2017b; Kamil et al., 2018; Alblooshi et al., 2022); thus the disease management capacity of BCAs heavily relies on uncontrollable environmental conditions (Ons et al., 2020).

Actinobacteria are a phylum of Gram-positive bacteria, which can be terrestrial or aquatic (Locci and Sharples, 1984; Lewin et al., 2016). The genus *Streptomyces* is a unique subgroup of actinobacteria that are known as prolific producers of antibiotics and bioactive secondary metabolites (Goodfellow and Williams, 1983; Barka et al., 2016; Lewin et al., 2016). Many species of *Streptomyces* exhibit biological control potential against phytopathogenic fungi through multiple mechanisms, including the production of antibiotics, hyperparasitism, and induction of plant resistance response (Tamreihao et al., 2016; Saeed et al., 2017a; Al Hamad et al., 2021; Alblooshi et al., 2022). Such an environmentally-friendly strategy has recently received renewed attention to control plant diseases and increase crop production.

Although several reports have used beneficial fungi (e.g., *Trichoderma* and *Chaetomium* species) as BCAs to control date palm pathogens (Soytong et al., 2005; Sánchez et al., 2007; Ammar, 2011; Nishad and Ahmed, 2020), no rhizosphere actinobacterial isolates have been identified against SDS. The endophytic *Streptomyces coeruleoprunus*, on the other hand, has been considered a biocontrol potential against *F. solani* on date palm trees in the UAE (Alblooshi et al., 2022). To avoid the worst impacts of black scorch disease, the rhizosphere isolate, *Streptomyces globosus* UAE1 has been successfully tested to control *T. punctulata* on date palm (Saeed et al., 2017a).

Previous studies showed that actinobacteria are important microorganisms in rhizosphere soils of the UAE (El-Tarabily et al., 2008; Mathew et al., 2020), and demonstrated an antagonism to phytopathogenic fungi including *T. punctulata* (Saeed et al., 2017a). The objectives of the present study were to: (i) isolate actinobacteria and test their abilities *in vitro* to produce antifungal metabolites and cell wall degrading enzymes (CWDEs) to inhibit *F. solani* DSM 106836; (ii) select the most promising isolate(s) possessing 1-aminocyclopropane-1-carboxylic acid (ACC) deaminase (ACCD) activities; and (iii) evaluate the antifungal activities of the identified ACCD-producing and non-ACCD-producing actinobacterial isolates against SDS on date palm seedlings in the greenhouse. Our results demonstrated that the ACCD-producing *Streptomyces violaceoruber* UAE1 can lessen the disease impact compared to the ACCD-non-producing isolate *Streptomyces tendae* UAE1 (*St*), thus, enhancing the resistance of date palm to *F. solani*. Our aim was to discover microbial resources (e.g., actinobacteria) from extreme environments to provide plant protection for sustainable agriculture.

## Materials and methods

### Cultivation of pathogen and isolation of actinobacteria

The fungus *F. solani* (DSM 106836), previously identified by Alwahshi et al. (2019), was subcultured on fresh potato dextrose agar (PDA; Lab M Limited, Lancashire, United Kingdom) plates (pH 6.0) and the plates were incubated at 28°C.

In order to isolate actinobacteria, five rhizosphere soil samples were randomly collected from healthy date palm trees (depth of ∼25 cm) of Mutaredh Oasis (24.22°N, 55.74°E) in Al-Ain city, UAE. The rhizosphere soils were air-dried for 4 days at 28°C (Williams et al., 1972). The soil dilution plate method (Johnson and Curl, 1972) using inorganic salt starch agar (ISSA; Küster, 1959) with specific soil pre-treatments (Hayakawa and Nonomura, 1987) was used to isolate streptomycete actinobacteria (SA). According to Nonomura and Ohara (1969), soil suspension was treated with a solution containing 6% of yeast extract (YE) (Sigma-Aldrich Chemie GmbH, Taufkirchen, Germany) and 0.05% of sodium dodecyl sulfate (SDS) (Sigma-Aldrich) at 40°C for 20 min, and subsequently diluted in water. This step was executed to increase actinobacterial populations and reduce the number of non-actinobacterial isolates.

To isolate non-streptomycete actinobacteria (NSA) from the rhizosphere, the following methods were implemented: (i) the use of polyvalent *Streptomyces* phages (Kurtböke et al., 1992); and (ii) the soil dry heat method (Nonomura and Ohara, 1969). These methods were used to reduce the abundance of SA and increase the dominance of NSA on isolation plates. For the polyvalent *Streptomyces* phages, the stock phage suspension was prepared by combining high-titer suspensions (×10^12^ plaque-forming units ml^–1^) of two different phages, and the stock suspension was then used to treat soil suspensions (10 g) in dilution tubes (five replicates). Actinobacterial colonies were isolated on ISSA plates which were dried in a laminar flow cabinet for 20 min and incubated at 28°C in dark for 2 weeks. Control treatments are those inoculated plates with soil dilutions that were not treated with the polyvalent phages. For the dry heat method, 10 g of each soil (five replicates) were heated at 120°C for 1 h. One gram of the heat-treated soil was added to 10 ml of water and vortexed for 5 min. A series of 10^–2^, 10^–3^, and 10^–4^ dilutions were prepared in sterile distilled water. Treatments consisting of unheated soil were used as a control. On arginine vitamin agar (AVA) plates, colonies of actinobacterial were spread and isolated (Nonomura and Ohara, 1969). AVA plates were air-dried and incubated at 28°C in dark for 2 weeks to encourage NSA growth.

Colonies of SA and NSA [log_10_ colony forming units (CFU) g dry soil^–1^] were purified on oatmeal agar plates (ISP-3 medium) amended with 0.1% of yeast extract (OMYEA; Küster, 1959) and were identified as previously described by Cross (1989). To distinguish between SA and NSA colonies, morphological criteria and the presence/absence of aerial mycelium, spore formation in aerial and substrate (vegetative) mycelia, and the stability of substrate mycelia were determined (Cross, 1989).

### *In vitro* bioassays for antifungal activities of actinobacterial isolates

All SA and NSA isolates were evaluated according to the secretion of diffusible antifungal metabolites against *F. solani* using the cut-plug method (Pridham et al., 1956). Isolates were inoculated on fish meal extract agar (FMEA) plates and incubated at 28°C in dark for 7 days (El-Tarabily et al., 1997). Plugs (11-mm) from cultures growing on FMEA were transferred to PDA plates seeded with *F. solani* that were kept at 28°C in dark for 5 days. The diameters of the inhibition zone (mm) for each isolate (eight plates) were determined.

Cultures of each isolate were also tested for their production of volatile compounds (VCs) on FMEA (Payne et al., 2000) at 28°C in dark for 10 days. FMEA plates were also inoculated with a 5-mm mycelial plug of the pathogen. After the lid removal, plates of *F. solani* were inverted over the actinobacterial plates (eight plates/isolate), taped together using Parafilm, and incubated at 28°C in dark for 7 days. Non-inoculated plates with any isolate served as a control. The colony diameter of *F. solani* (mm) was measured and compared to that of the control.

In addition, all actinobacterial isolates were evaluated for CWDEs synthesis by measuring the clearing zones (mm) around and beneath the actinobacterial colonies using *F. solani* mycelial fragment agar (MFA; Valois et al., 1996). Only highly active CWDEs-producing isolates showing large diameters of clearing zones (>30 mm) were selected, whereas others were discarded. Chitinase activity on colloidal chitin agar (CCA) plates of each isolate (eight plates/isolate) was also determined. An efficient method of preparing colloidal chitin from crab shell chitin (Sigma-Aldrich) was developed (Hsu and Lockwood, 1975). CCA plates were incubated at 28°C in dark for 7 days and the clearing zone (mm) produced by the individual isolates around and beneath the colonies was measured (Gupta et al., 1995).

An isolate was considered a siderophore producer when a yellow-orange halo zone around the colony developed on chrome azurol S (CAS) agar plates inoculated with a particular BCA and incubated at 28°C in dark for 3 days (Schwyn and Neilands, 1987).

The promising isolates were also tested for the synthesis of hydrogen cyanide (HCN) by adopting the method of Bakker and Schippers (1987). Each actinobacterial strain was grown on tryptic soy agar (TSA; Lab M Limited) plates containing 4.4 g l^–1^ of glycine and was incubated at 28°C for 5 days. A Whatman filter paper No. 1 soaked in 2% of sodium carbonate prepared in 0.5% of picric acid solution was placed on the top of the plate and incubated at 28°C for 5 days. The development of an orange-to-red color indicated the formation of HCN.

Only isolates showing strong inhibition against *F. solani* on FMEA plates (diffusible antifungal metabolite- and VC-producers), large clearing zones on MFA and CCA plates (CWDE-and chitinase-producers), and siderophore production were chosen for further analyses. The rest of the isolates showing no or weak inhibition and clearing zones of less than 30 mm as well as no production of siderophore or HCN were not considered.

### *In vitro* determination of ACC utilization and measurement of ACCD activity

The activity of ACCD was initially screened on Dworkin and Foster (DF) salt minimal agar medium with 3 mM of ACC (Sigma-Aldrich) as a sole N source (Dworkin and Foster, 1985). For positive control, the DF minimal medium was supplemented with 0.2% of (NH_4_)_2_SO_4_; and for the negative control, DF medium devoid of any N source was used. Growth of isolates on DF medium supplemented with ACC (DF-ACC agar) was assessed after incubation at 28°C in dark for 7 days and compared to controls. The ACCD producers were selected based on growth on DF-ACC plates as an indicator of the efficiency of the selected isolates to utilize ACC.

The potential activities of BCAs were also quantitatively determined by growing them in inorganic salt starch broth (ISSB; Küster, 1959) at 28°C in dark for 5 days. Spores were harvested, inoculated onto DF-ACC broth (eight independent flasks/isolate), and incubated at 28°C in dark for 5 days on a rotary shaker (Model G76, New Brunswick Scientific, NJ, United States) at 250 rpm. Cells were collected, resuspended in 0.1 M Tris-HCl (pH 8.5), followed by three cycles of freeze/thaw (placing in liquid-N for 1 min, and immersing in a warm water bath for 5 min). The activity of ACC deamination was assayed by measuring α-ketobutyrate formed from the cleavage of ACC (Honma and Shimomura, 1978). One unit of enzyme is the forming activity of 1 μmol of product in 1 min at 28°C. Protein concentration was determined using the method of Bradford (1976).

### Phylogenetic analysis and morphological identification of the most promising BCAs

According to Locci (1989), BCA isolates #6 and #26 were identified at the species level. For genomic DNA extraction, cultures were grown in 20 ml of TSB in 50-ml Erlenmeyer flasks for 7 days and then centrifuged for 5 min at 12,000 × *g* (Centra 4 model centrifuge; International Equipment Company, Woonsocket, RI, United States). Mycelial pellets were resuspended in 500 μl of 5 M NaCl and transferred to a 2-ml Eppendorf tube. Cells were centrifuged at 12,000 × *g* for 30 s, and the pellet was resuspended in 1 ml of 10 mM Tris-HCl, l mM EDTA (TE; pH 7.5) containing 20 mg ml^–1^ of each lysozyme and RNase A and incubated at 37°C for 1 h. Following incubation, 250 μl of 0.5 M EDTA, 250 μl of TE containing 5 mg ml^–1^ of proteinase K, and 100 μl of 10% SDS were added to each tube and incubated at 37°C for 1 h. Tubes were mixed by inversion after adding 250 μl of 5 M NaCl. Immediately, 200 μl of cetyltrimethylammonium bromide (CTAB) solution (10% CTAB + 0.7 M NaCl) was added and tubes were heated in a water bath at 65°C for 10 min. After centrifugation, using an Eppendorf model 5415 centrifuge at 12,000 × *g* for 5 min, the supernatant solution was transferred to a new 2-ml microcentrifuge tube. One-third of the volume of phenol-chloroform was added, and the phases were mixed and centrifuged at 14,000 × *g* for 5 min. The aqueous phase was transferred to a new tube and DNA was precipitated with isopropanol. After centrifugation, the pellet was rinsed with 70% ethanol, dried, and redissolved in 200 μl of TE.

The two isolates were identified based on their 16S rRNA gene sequence analysis. The amplified products of 16S rRNA (∼1,520 bp) from gDNA obtained from bacterial cultures by PCR were sequenced using the primers described by Rainey et al. (1996): 907R (5′-CCGTCAATTCATTTGAGTTT-3′); 803F (5′-ATTAGATACCCTGGTAG-3′) and 357F (5′-TACGGGAGGCAGCAG-3′). The reaction mixture contained ∼50 ng of DNA, *Ex*Taq PCR buffer, 1.5 mM of MgCl_2_, 10 mM of deoxynucleoside triphosphate mixture, 50 pmol of each primer, and 0.5 U of Ex*Taq* polymerase. PCR conditions consisted of an initial denaturation at 95°C for 3 min; 28 cycles at 95°C for 1 min (denaturation), 55°C for 1 min (annealing), and 72°C for 2 min (extension), followed by a final 5-min extension at 72°C. All sequencing reactions and phylogenetic analyses were carried out by Deutsche Sammlung von Mikroorganismen und Zellkulturen GmbH (DSMZ), Braunschweig, Germany.

Briefly, pairwise sequence similarity using 16S rRNA gene sequence was determined. For the phylogenetic analyses, the reference strain was selected from the top hits of the determination using GenBank BLAST^1^. To predict the species of isolates, the neighbor-joining method implemented in Molecular Evolutionary Genetics Analysis 7.0 (MEGA7) software (Saitou and Nei, 1987; Kumar et al., 2016) was used. Bootstrap values were calculated with 500 resampled datasets.

Scanning electron microscopy (SEM) was carried out using the Philips XL-30 SEM (FEI Co., Eindhoven, Netherlands) to examine the morphology of spore chains and surfaces. The SEM analysis was determined following the growth on ISP3 medium (Shirling and Gottlieb, 1966) at 28°C for 14 days.

### *In vivo* greenhouse experiments

*In vivo* bioassays were performed to determine the efficacy of BCAs on date palm seedlings in soil infested with *F. solani*. In greenhouse Experiment (1), date palm seedlings were transplanted in soil colonized with each of the ten potential isolates showing the strongest production of diffusible antifungal metabolites and CWDEs (see Section “*In vitro* Bioassays for Antifungal Activities of Actinobacterial Isolates”) of which four were ACCD producers (isolates #26, #43, #46, and #50) and six were not ACCD producers (#6, #7, #33, #40, #42, and #44) (see Section “*In vitro* Determination of ACC Utilization and Measurement of ACCD Activity”). Similarly, BCA1 (isolate #6) and BCA2 (#26) were further tested on date palm seedlings cultivated in *F. solani*-infested soil (Experiment 2).

In both experiments, BCA applications and pathogenicity tests were conducted on 6-month-old date palm (cv. Barhi) seedlings obtained from the Date Palm Development Research Unit, UAE University, UAE. To ensure rhizosphere/root colonization by the BCAs, soils were initially treated with the individual BCA for 7 days prior to *F. solani* inoculation.

To prepare the pathogen inoculum, millet (*Panicum miliaceum* L.) seeds were prepared by soaking 25 g of seeds to 40 ml of distilled water in 250 ml of Erlenmeyer flasks which were autoclaved on three consecutive days at 121°C for 45 min (El-Tarabily et al., 2000). Sterilized-millet seeds were inoculated with 20 agar plugs from the actively growing margins of an *F. solani* colony and incubated at 28°C for 2 weeks in dark. To ensure uniform colonization, the flasks containing 1 × 10^6^ spores ml^–1^ of *F. solani* were shaken periodically. The control consisted of colonized and autoclaved millet seeds. Before use, small amounts of the control and colonized millet seeds were plated onto PDA to confirm the absence or presence of *F. solani*.

The antagonist inoculums were prepared by placing 50 g of moist oat bran into 500 ml Erlenmeyer flasks and autoclaving at 121°C for 20 min on three successive occasions as described by El-Tarabily et al. (2000). A 25-ml spore suspension, adjusted to 10^8^ cfu ml^–1^ by using a hemocytometer (Agar Scientific Limited, Essex, United Kingdom), of each antagonist was aseptically inoculated into the oat-bran and incubated at 28°C for 3 weeks in dark. To ensure uniform colonization, Erlenmeyer flasks were shaken frequently. Similarly colonized and autoclaved oat bran was used as a control. The colonized oat bran and the control oat bran were both suspended in 50 ml of sterile distilled water prior to use. To confirm the presence or absence of antagonists, an aliquot of the suspension (0.2 ml) was spread onto OMYEA plates which were incubated at 28°C for 7 days weeks in dark.

In Experiment (2), the aim was to test the efficacy of BCA1 and BCA2 prior to *F. solani* infection on date palm. In this experiment, six treatments were applied:

(i) Healthy control (C): seedlings cultivated in the soil without the pathogen, *F. solani*;
(ii) Diseased control (*Fs*): seedlings cultivated in the soil with *F. solani*-colonized millet seeds only;
(iii) *St*: seedlings cultivated in the soil colonized with *S. tendae* UAE1 (BCA1; non-ACCD-producing isolate #6) without *F. solani*;
(iv) *St* + *Fs*: seedlings cultivated in the soil colonized with *S. tendae* UAE1 followed by *F. solani* inoculation;
(v) *Sv*: seedlings cultivated in the soil colonized with *S. violaceoruber* UAE1 (BCA2; ACCD-producing isolate #26) without *F. solani*; and
(vi) *Sv* + *Fs*: seedlings cultivated in the soil colonized with *S. violaceoruber* UAE1 followed by *F. solani* inoculation.

To carry out the pathogenicity test and biological control trials, the soil was collected from the same farm described above (see Section “Cultivation of Pathogen and Isolation of Actinobacteria”) and air-dried before being passed through a 3-mm mesh sieve. The antagonist-colonized oat bran inoculum (1% weight of colonized oat bran inoculum/weight of air-dried non-sterile soil) was dispersed through the soil using a cement mixer. The BCA-colonized soil was added into 4 kg of plastic-free draining plant pots and left for 7 days on a bench in an evaporative-cooled greenhouse (15-h day/9-h night; temperature = 25 ± 2°C; relative humidity = 60 ± 5%; photosynthetic photon flux density = 700 μmol m^–2^ s^–1^). The pathogen inoculum (1% weight of colonized millet seed inoculum/weight of air-dried non-sterile soil) was added to each pot and dispersed through the soil by the same method as that of the BCAs. Date palm seedlings were planted in pots containing all the combinations described above and grown randomly on a bench in the greenhouse, and the pots were watered every 2 days.

For each treatment, eight pots (one seedling pot^−1^) were arranged in a completely randomized design. Plants were kept in the greenhouse for 35 days post-treatment (dpt) for Experiments 1 and 2. Disease severity index was recorded for SDS symptoms at 35 dpt (Experiment 1) and at 15 and 35 dpt (Experiment 2) using a scale of 0–5: 0 = no apparent symptoms, 1 = 1–10% necrotic or white area in leaves or rotting in roots, 2 = 11–25%, 3 = 26–50%, 4 = 51–75%, and 5 = 76–100% (Alblooshi et al., 2022). For disease symptoms/recovery of diseased plants, conidial counts of *F. solani* were recorded at 35 dpt (Experiment 2). Conidia were harvested from the affected root tissues of eight seedlings treatment^−1^ in 5 ml of water, and counted using a hemocytometer (Agar Scientific Limited, Essex, United Kingdom) as previously described (Alwahshi et al., 2019). All *in vivo* experiments were conducted on three independent occasions to confirm reproducibility. Two independent greenhouse runs (8 plants per treatment per run) were pooled for statistical analysis (*n* = 16 plants per treatment) after confirming similar results across runs. At the end of the experiment, the fungus was re-isolated from tissues with disease symptoms attempting to fulfill Koch’s Postulates.

### Measurements of endogenous ACC in plant tissues with high-pressure liquid chromatography

At the end of greenhouse Experiment (2), endogenous ACC contents were determined from date palm tissues. Pieces of roots and shoots (each was 10 cm in length) were collected. According to Lanneluc-Sanson et al. (1986), derivatization of ACC was carried out by adding phenylisothiocyanate (Sigma-Aldrich), and the subsequent separation and quantification of the resulting phenylthiocarbamyl-ACC by reverse-phase high-pressure liquid chromatography (HPLC). Phenylthiocarbamylation of ACC, and other amino acids, in date palm extracts was completed within 20 min at 25°C. After removing solvents and reagents, the phenylthiocarbamyl derivatives were separated on an octadecyl reverse-phase column, eluted with a mixture of acetonitrile and sodium acetate buffer (pH 4.6), and monitored with a UV-detector set at 254 nm. After removing solvents and reagents, 10 μl of the resulting phenylthiocarbamyl derivatives were separated on an octadecyl 10-μm reverse-phase column (Waters Associates μBondapak C_18_, 4 mm × 30 cm), eluted with a mixture of acetonitrile and sodium acetate buffer (pH 4.6) in a Waters Associates liquid chromatograph and monitored with a differential 254 nm-UV detector. An analysis of date palm extract was achieved in 25 min and detected quantities as low as 1 pmol. Eight independent replicate samples were analyzed.

The concentration of ACC was obtained by the comparison of the peak area in the unknown sample with that of the corresponding area obtained with the authentic samples of a known concentration (Sigma–Aldrich).

### Quantitative production of antifungal compounds and CWDEs

Only the non-ACCD-producing BCA1 and ACCD-producing BCA2 were further assessed for the production of diffusible antifungal metabolites against *F. solani* using the cup plate method (Bacharach and Cuthbertson, 1948). Erlenmeyer flasks (250 ml) containing 50 ml of sterile fish meal extract broth (FMEB; El-Tarabily et al., 1997) were inoculated with 1 ml of 10% glycerol suspension of each BCA (∼10^8^ cfu ml^–1^) and incubated at 200 rpm on a gyratory shaker (Model G76, New Brunswick Scientific-Edison, NJ, United States) in dark at 28°C for 5 days. The suspensions from each flask were centrifuged at 8,000 × *g* for 30 min. The crude culture filtrate (supernatant) was filtered using 0.22 μm Millipore membranes (Millipore Corporation, MA, United States) and stored at 4°C. To prepare *F. solani*-seeded PDA plates, inocula were prepared according to the same method as for the cut-plug technique above. A sterilized 11-mm cork borer was used to cut the centers of the freshly seeded PDA plates with *F. solani*. By using a sterilized syringe, aliquots (0.3 ml) of the filter-sterilized crude culture filtrate were injected into the wells. The diameter of inhibition zones was determined for the two selected BCAs after 5 days of incubation in dark at 28°C.

A dialysis membrane (Type 45311; Union Carbide Corporation, IL, United States) overlay technique on FMEA or CCA plates (Gupta et al., 1995) was also performed to assay inhibition of *F. solani* by BCAs as previously recommended (El-Tarabily et al., 1997). Briefly, the membrane surface was inoculated with the BCA by evenly streaking cells/spores of a 7-day old culture of BCAs grown on OMYEA. Then, the FMEA or CCA plates were incubated at 28°C in dark for another 10 days. The membranes of adhering colonies were subsequently removed from the agar plates and the center of the plate was inoculated with a 5-mm disk of *F. solani* culture grown for 7 days on FMEA at 28°C in dark. The diameter of *F. solani* colony (mm) was measured after 8 days and compared to that of FMEA or CCA plates with the growing pathogen without any BCA (control). In case no pathogen grew from the agar plugs, these plugs were transferred to a fresh PDA plate, and incubated at 28°C for 5 days. If the pathogen did not grow from the plug, the diffused metabolites were fungicidal; however, if otherwise, these metabolites were considered fungistatic.

The production of chitinase and β-1,3-glucanase by the BCAs (Singh et al., 1999) was quantitatively determined using the minimal synthetic medium (Tweddell et al., 1994) amended with 2 mg ml^–1^ of either colloidal chitin or laminarin (Sigma-Aldrich), respectively. Chitinase-specific activity was calculated by measuring the release of *N*-acetyl-D-glucosamine (NAGA) from colloidal chitin. One unit (U) of chitinase activity was defined as the amount of enzyme releasing 1 μmol of NAGA mg^–1^ protein h^–1^ (Reissig et al., 1955) under the assay condition. The specific activity of β-1,3-glucanase was determined by measuring the amount of reducing sugars liberated from laminarin using dinitrosalicylic acid (DNS) solution (Miller, 1959). One U of β-1,3-glucanase activity was defined as the amount of enzyme that produced 1 μmol of reducing sugar mg^–1^ protein h^–1^ (Miller, 1959) under the above conditions. Protein concentration was determined by the Folin phenol reagent method (Lowry et al., 1951), using bovine serum albumin as standard.

### Evaluation of crude extracts from culture filtrates of BCAs on *F. solani*

Filter-sterilized crude culture filtrates of BCA1 and BCA2 using FMEB or colloidal chitin broth (CCB) (Gupta et al., 1995) were proportionally poured in PDA plates. The medium was inoculated with a 5-mm diameter agar plug of *F. solani* mycelium that was placed upside-down. The colony diameter (mm) of the pathogen was measured after 10 days at 28°C. Inhibition of mycelial growth was also calculated upon mixing crude culture filtrates with potato dextrose broth (PDB; Lab M) inoculated with a 5-mm diameter agar plug of *F. solani* (Lorito et al., 1993) after 10 days of incubation in dark at 28°C.

To determine the mycelial dry weight, the tested mycelial contents of each culture were placed in a beaker containing boiling distilled water for 4 min as previously described by Suiton Iii and Starzyk (1972). The contents were then filtered through a Buchner funnel by using Whatman no. 114 filter paper. The contents were washed and the resultant mycelial mat on the filter paper with a constant vacuum of 15 psi was maintained throughout the working procedure. After the final wash, the sample was aspirated, and dry weights were obtained by placing the samples in a vacuum desiccator for 6 days. Then, the dry weight of mycelia was recorded for each sample.

Using Nikon-Eclipse 50i light microscope at 40× (Nikon Instruments Inc., NY, United States), the percentage of spore germination and the average germ tube length of *F. solani* after 24 h in PDB (Lorito et al., 1993) were determined. Briefly, tubes containing PBD, actinobacterial isolates, enzymes, and the fungus (treatment) or water (control) were incubated at 25°C for 24 h. The percentage of the first 100 spores seen on the microscope slide was considered as the percentage of germinating conidia, and the length of 20 germ tubes was measured and averaged. Observations with an oil immersion lens (100×) were essential to examine the effects of the crude culture filtrates of BCAs on the hyphal morphology of *F. solani* (Sneh, 1981). For all control treatments, *F. solani* mycelium was incorporated with non-inoculated filter-sterilized FMEB or CCB.

### Statistical analysis

*In vitro* evaluation of BCAs against *F. solani*, data were analyzed using analysis of variance (ANOVA) and Duncan’s multiple range test at 5% level of significance. These experiments were repeated thrice using eight plates treatment^−1^ for each time with similar results.

The two greenhouse experiments were independently repeated twice, and the obtained data were combined and analyzed. Each experiment included eight plants per treatment, resulting in a total of 16 plants per treatment. Data are presented as mean ± SE. Data were first analyzed using a one-way ANOVA, and treatment means were then compared using Duncan’s multiple range test at a significance level of P< 0.05. Mean values followed by the same letter are not significantly different, whereas those followed by different letters are significantly different from each other. For all statistical analyses, SAS Software version 9 (SAS Institute Inc., NC, United States) was used.

## Results

### Isolation and screening of actinobacteria with antifungal properties

From the 54 actinobacteria, 34 SA (62.9%) and 20 NSA (37.1%) strains were isolated on ISSA plates from the rhizosphere soil of healthy date palm trees. Prior to isolation, polyvalent *Streptomyces* phages and dry heat methods were effectively used to introduce diverse rare actinobacterial isolates, i.e., NSA. Consequently, the numbers of SA were significantly (*P* < 0.05) reduced, but the numbers of NSA significantly (*P* < 0.05) increased by 66.8 and 70.3% by polyvalent *Streptomyces* phages and dry heat pretreatment, respectively (Supplementary Table 1 and Supplementary Figure 1). Besides the dominant *Streptomyces*, rare actinobacteria of the genera *Actinomadura*, *Actinoplanes*, *Dactylosporangium*, *Kribbella*, *Microbispora, Micromonospora*, *Microtetraspora, Nocardia*, *Nocardiopsis*, *Pseudonocardia*, *Rhodococcus*, and *Streptosporangium* were also isolated.

Next, two *in vitro* screenings were simultaneously carried out to determine the production of diffusible antifungal metabolites and CWDEs. Our results showed that 25 isolates (12 SA and 13 NSA) showed strong production of both diffusible antifungal metabolites and CWDEs against *F. solani* (Table 1 and Supplementary Figure 2). Isolates belonging to SA (#2, #6, #9, #17, #22, #26, #29, #33, #37, #41, #42, and #50) and NSA (#7, #12, #16, #20, #38, #40, #43, #44, #46, #49, #51, #53, and #54) produced large inhibition and clearing zones (>30 mm) on FMEA and MFA, respectively, were further selected for subsequent experiments (Table 1). The rest of the isolates showing inhibition zones on FMEA or clearing zones on MFA less than 30 mm were excluded.

**TABLE 1:** *In vitro* antagonistic and ACCD enzymatic activities by actinobacterial isolates against *Fusarium solani.*

Taxa	Species	Isolate	Inhibition diameter^a^	Clearing diameter^b^	Production of		ACCD activity^c^
					
			(mm)		VC	Siderophore	nmol α-ketobutyrate mg^–1^ protein h^–1^
	*F. solani* (pathogen)		0.0	0.0	−	−	0.00 a
	#13 (positive control)		0.0	0.0	−	−	0.00 a
SA	*Streptomyces*	#2	56.1 ± 2.4 d	50.3 ± 2.0 c	−	−	ND
		#6	58.3 ± 2.9 d	59.2 ± 3.1 d	+	+	0.00 a
		#9	47.2 ± 1.2 c	57.3 ± 1.8 d	−	+	ND
		#17	45.2 ± 0.9 bc	43.2 ± 1.1 b	−	−	ND
		#22	31.6 ± 0.6 a	44.2 ± 1.3 b	+	−	ND
		#26	58.9 ± 2.2 d	58.0 ± 2.9 d	+	+	548.28 ± 27.8 e
		#29	30.2 ± 0.5 a	30.8 ± 0.7 a	+	−	ND
		#33	41.9 ± 2.3 b	31.4 ± 0.6 a	+	+	0.00 a
		#37	58.7 ± 2.8 d	31.8 ± 0.9 a	−	−	ND
		#41	44.5 ± 1.4 b	57.7 ± 2.3 d	−	+	ND
		#42	43.3 ± 3.4 b	42.4 ± 2.1 b	+	+	0.00 a
		#50	58.2 ± 1.9 a	46.6 ± 2.4 bc	+	+	46.72 ± 11.3 b
NSA	*Actinomadura*	#7	57.8 ± 2.0 d	47.5 ± 1.7 bc	+	+	0.00 a
		#20	30.6 ± 0.4 a	56.8 ± 1.8 d	+	−	ND
	*Actinoplanes*	#40	33.0 ± 0.4 a	32.7 ± 0.6 a	+	+	0.00 a
		#43	42.6 ± 1.6 b	42.2 ± 0.8 b	+	+	368.66 ± 18.6 d
		#53	33.0 ± 1.2 a	57.7 ± 2.1 d	−	−	ND
	*Dactylosporangium*	#49	58.0 ± 2.6 d	58.8 ± 1.2 d	−	−	ND
	*Microbispora*	#12	31.7 ± 0.8 a	44.4 ± 1.4 b	−	+	ND
	*Micromonospora*	#16	31.5 ± 0.2 a	31.7 ± 0.8 a	+	−	ND
		#38	43.9 ± 1.1 b	31.2 ± 0.8 a	−	−	ND
		#46	57.2 ± 2.2 d	43.3 ± 1.2 b	+	+	141.24 ± 8.8 c
		#51	32.3 ± 0.6 a	31.2 ± 0.5 a	−	+	ND
	*Microtetraspora*	#44	32.2 ± 0.7 a	42.2 ± 1.0 b	+	+	0.00 a
	*Streptosporangium*	#54	42.0 ± 0.9 b	45.1 ± 1.3 bc	−	+	ND

^a^Production of diffusible antifungal metabolites active against F. solani using the cut-plug method.

^b^Production of CWDEs on MFA.

^c^Production of ACCD on DF-ACC medium for 5 days at 28 ± 2°C.

For all in vitro experiments, values are means ± SE of eight replicates. Values within each column, followed by the same letter are not significantly (P > 0.05) different according to Duncan’s multiple range test.

Isolate #13 is a non- antifungal-, non-CWDE- and non-ACCD-producing positive control. Isolates #6 and #26 represent the ACCD-non-producing Streptomyces tendae UAE1 (BCA1) and the ACCD-producing Streptomyces violaceoruber UAE1 (BCA2), respectively.

SA, streptomycete actinobacteria; NSA, non-streptomycete actinobacteria; VC, volatile compounds; ACC, 1-aminocyclopropane-1-carboxylic acid; ACCD, ACC deaminase; CWDEs, cell-wall-degrading enzymes; MFA, mycelial fragment agar; DF, Dworkin and Foster’s salts minimal broth medium; BCA, biological control agent; +, producing; −, not producing; ND, not determined.

Considering the multiple mechanisms of antifungal effects, the above-mentioned isolates were checked if they could produce VC, siderophores, and HCN. Only ten isolates of SA and NSA (#6, #7, #26, #33, #40, #42, #43, #44, #46, and #50) were found to share the production of diffusible antifungal metabolites and CWDEs in addition to the production of VC (Table 1) on FMEA and siderophores on CAS agar plate (Table 1 and Supplementary Figure 3). None of the isolates, on the other hand, produced HCN. Isolate #13 (the non-antifungal/CWDE/ACCD producer) was used as a positive control in all *in vitro* tests. These potent ten isolates were further selected for subsequent experiments. Our data suggest that the actinobacteria (SA and NSA) isolated from the rhizosphere soils and showing multiple modes of antagonism against *F. solani* can be effective to manage SDS in the UAE.

### Effect of ACCD activities produced by the rhizospheric actinobacterial isolates

In addition to the tested antifungal properties, the enzymatic ACCD activities of the ten potential BCAs on DF-ACC plates were quantitatively determined (Supplementary Figure 3). Four isolates (#26, #43, #46, and #50) were considered ACCD producers, albeit the variation in ACCD levels among them (Table 1). On the other hand, six isolates (#6, #7, #33, #40, #42, and #44) were non-ACCD producers. These isolates were further investigated under greenhouse conditions.

**FIGURE 1:**
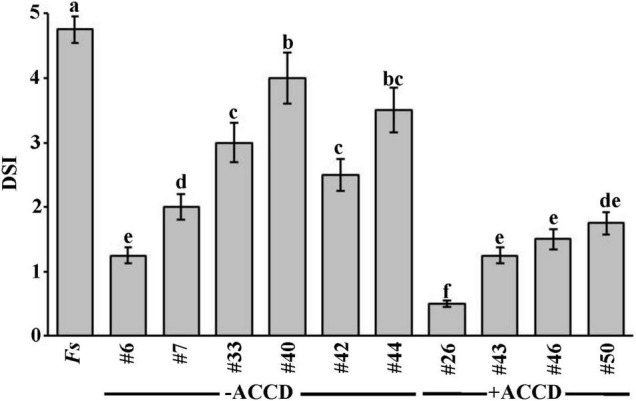
*In vivo* effect of selected BCAs on date palm seedlings inoculated with *Fusarium solani* (Experiment 1). Disease severity index after the recovery of affected date palm seedlings from the fungal pathogen, *F. solani*, at 35 dpt with the BCA under greenhouse conditions. DSI was based on a scale of 5:0 = no infection, 1 = 1–10, 2 = 11–25, 3 = 26–50, 4 = 51–75, and 5 = 76–100% damage including necrosis, white area in leaves or rotting in roots. Values with different letters are significantly different from each other at *P* < 0.05. Bars represent standard errors. Seedlings (*n* = 16) were cultivated in soil colonized with each BCA isolate 7 days prior to soil infestation with *F. solani*. Isolates #6 and #26 represent the ACCD-non-producing *Streptomyces tendae* UAE1 (BCA1) and ACCD-producing *Streptomyces violaceoruber* UAE1 (BCA2), respectively. BCA, biological control agent; *Fs*, seedlings cultivated in soil infested with *F. solani* only; dpt, days post treatment; DSI, disease severity index; ACCD, 1-aminocyclopropane-1-carboxylic acid deaminase.

The *in vivo* Experiment (1) was carried out on date palm seedlings cultivated in soil colonized with *F. solani* using the potential ten antagonists. For that reason, we visually estimated DSI on seedlings to assess their recovery from SDS caused by *F. solani* after the application of BCAs. Date palm seedlings cultivated in soil infested with *F. solani* without any BCA treatment, designated as *Fs*, did not recover from the disease by the end of Experiment 1 (Figure 1).

In general, there was a significant (*P* < 0.05) difference in DSI among all treatments on diseased seedlings (Figure 1). Thus, seedlings that were treated with ACCD-producing isolates demonstrated lower DSI than those treated with ACCD-non-producing isolates (except of #6) or *Fs* (control) treatment. On the other hand, plants treated with individual ACCD-non-producing isolates for 7 days before inoculation with the pathogen significantly (*P* < 0.05) showed lesser DSI than in control plants. Thus, isolate #6 was the best among the tested ACCD-non-producing isolates, and was termed BCA1.

Similarly, plants grown in soils amended with any of the ACCD-producing isolates infested with *F. solani* were compared for their recovery. Even though the ACCD-producing isolates #43, #46, and #50 showed a comparable reduction of DSI on diseased seedlings, they did not reach to the same level of reduction obtained by isolate #26 at 35 dpt (Figure 1). Isolate #26, also labeled as BCA2, was relatively superior over the others; thus, its preventive application dramatically reduced DSI resulting in minimal disease effect and relatively healthy-grown seedlings. Our preliminary data suggest that ACCD production might be a major mechanism that BCAs utilize to offer promise for the improvement of plant health in response to biotic stresses.

### Identification of the promising BCAs

In order to identify BCA1 (isolate #6) and BCA2 (isolate #26) to the species level, the nucleotide sequence of their 16S rRNA gene was determined. The resulting sequences were deposited in NCBI^2^ under GenBank accession numbers: OL356342 for BCA1, and OL356341 for BCA2.

In comparison with other 16S rRNA gene sequences obtained from the GenBank database, the 1,520-bp sequence of BCA1 showed 99.9% similarity with that in *Streptomyces tendae* ATCC 19812^T^ (D63873) and *Streptomyces violaceorubidus* LMG 20319^T^ (AJ781374; Supplementary Figure 4). The rest of the *Streptomyces* spp. showed less similarity with our studied strain. To obtain definitive identification of isolate #6, its pure culture was cultivated on ISP medium 3. After 14 days of cultivation, the isolate developed light gray aerial mycelium and yellow to greenish yellow substrate mycelium with the production of yellow pigment on the reverse side of cultures (Supplementary Figure 4). The configuration of the spore chains of the isolate was examined using SEM and our results revealed that spore chains belonged to section Spirales (closed spirals), consisting of 10–50 mature spores with smooth surfaces (Supplementary Figure 4). Together, this suggests that the outstanding isolate #6 can be identified as *S. tendae* (Ettlinger et al., 1958) strain UAE1.

**FIGURE 2:**
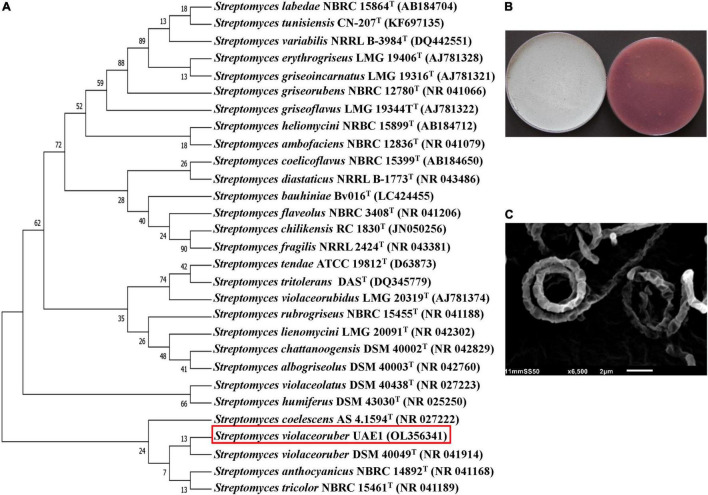
Taxonomic identification of the ACCD-producing *Streptomyces violaceoruber* UAE1 (BCA2). **(A)** The dendrogram showing the phylogenetic relationships between *S. violaceoruber* UAE1 (isolate #26; 1519 bp; OL356341) and other members of *Streptomyces* spp. on the basis of 16S rRNA sequences. **(B)** Gray aerial (left) and violet substrate mycelia with the production of violet pigment on the reverse side of cultures (right) growing on ISP3 medium supplemented with yeast extract; and **(C)** scanning electron micrograph (6,500×) of the section Spirales spore chains (closed spirals) with 10–50 smooth-surfaced spores/chain of *S. violaceoruber* UAE1. In **(A)**, numbers at nodes indicate percentage levels of bootstrap support based on a neighbor-joining analysis of 500 resampled datasets. GenBank accession numbers are given in parentheses. BCA, biological control agent; ACCD, 1-aminocyclopropane-1-carboxylic acid deaminase.

The 1,519-bp of 16S rRNA of the other antagonist (BCA2) showed 100% similarity with the nucleotide sequence of four *Streptomyces* spp., namely *S. violaceoruber* DSM 40049^T^ (NR041914), *S. anthocyanicus* NBRC 14892^T^ (NR041168), *S. tricolor* NBRC 15461^T^ (NR041189), and *S. coelescens* AS 4.1594^T^ (NR027222; Figure 2A). Consequently, its pure cultures were checked on ISP medium 3. It turned out that aerial mycelia mass color was gray with violet substrate mycelium and with the production of violet pigment on the reverse side of cultures (Figure 2B). The formation of spore chains belonged to the Spirales type (closed spirals) that consisted of 10–50 smooth-surfaced spores/chains on the aerial hyphae (Figure 2C). BCA2 was assigned as *S. violaceoruber* (Waksman and Curtis, 1916) Pridham, 1970 strain UAE1. It is worth mentioning that the suffix ‘UAE1’ for both Streptomyces species denotes the geographical origin of the isolates and is not intended as a unique strain identifier.

### Effects of *S. tendae* or *S. violaceoruber* on date palm seedlings in *F. solani*-infested soils (Experiment 2)

**FIGURE 3:**
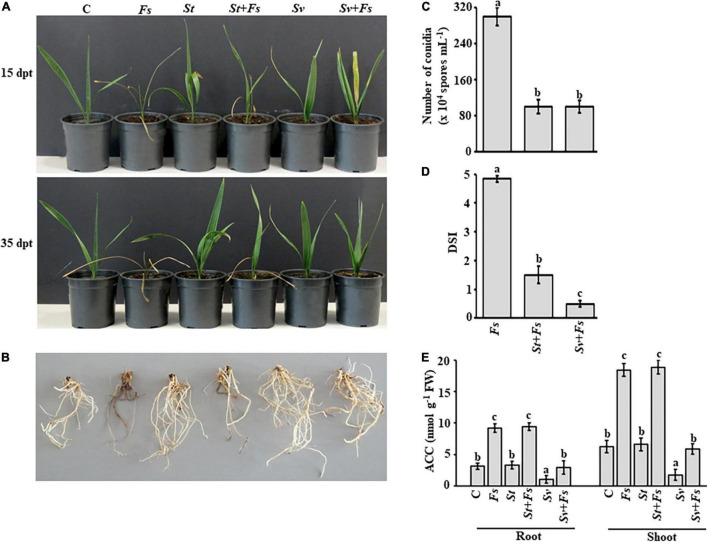
Effect of BCA treatments on date palm seedlings in soils infested with *Fusarium solani*. Preventive effect of BCA treatments on **(A)** seedlings and **(B)** roots in soil not-infested or infested with *F. solani* after 15 (upper panel) and 35 dpt (lower panel). Recovery of seedlings (*n* = 16) from pathogen infection was measured according to **(C)** the number of conidia; **(D)** DSI, **(E)** endogenous contents of ACC in the root and shoot tissues of inoculated date palm seedlings after BCA treatment after 35 dpt under greenhouse conditions. In **(D)**, DSI was based on a scale of 5:0 = no infection, 1 = 1–10, 2 = 11–25, 3 = 26–50, 4 = 51–75, and 5 = 76–100% damage including necrosis, white area in leaves or rotting in roots. In **(C–E)**, values with different letters are significantly different from each other at *P* < 0.05. Bars represent standard errors. C, seedlings grown in non-pathogen colonized soil (control); *Fs*, seedlings grown in soil infested with *F. solani* (diseased control); *St* or *Sv*, seedlings grown in soil colonized with either the non-ACCD-producing *Streptomyces tendae* UAE1 (BCA1; isolate #6) or the ACCD-producing *Streptomyces violaceoruber* UAE1 (BCA2; isolate #26), respectively; *St* + *Fs* or *Sv* + *Fs*, seedlings grown in soil colonized with either *S. tendae* UAE1 or *S. violaceoruber* UAE1, respectively, 7 days prior to incorporation of *F. solani*-millet seed based inoculum; ACC, 1-aminocyclopropane-1-carboxylic acid; ACCD, ACC deaminase; BCA, biological control agent; FW, fresh weight; dpt, days post treatment; DSI, disease severity index.

The efficacy of BCA1 and BCA2 against SDS was tested and compared with date palm (cv. Barhi) in a greenhouse pot study. Seedlings transplanted in soils infested with *F. solani* (*Fs*) displayed typical foliar and root symptoms of SDS. At the early stages, leaves developed chlorosis (yellowing), wilting, and mild necrosis, turning to brown, dryness, and death of plants at later stages (Figure 3A). Advanced foliar symptoms in plants removed from the soil were associated with a weak, rotted root system when compared to a healthy root system (Figure 3B).

Treatments of either *S. tendae* UAE1 (BCA1; ACCD-non-producing isolate) or *S. violaceoruber* UAE1 (BCA2; ACCD-producing isolate) exhibited varying degrees of SDS suppression on the above and underground parts of infected seedlings. We noticed that plants cultivated in soils infested with *F. solani* that were pre-treated with BCA1 (*St* + *Fs*) and BCA2 (*Sv* + *Fs*) started to recover at 15 dpt (Figure 3A). At 35 dpt, not only newly fresh leaves in the center of the crown emerged, but also the root system appeared healthy and strong (Figure 3B), confirming our *in vitro* results of the inhibitory effect of BCAs against *F. solani*. This is in contrast to *Fs* plants without BCA treatments. In general, the application of *S. violaceoruber* UAE1 was visually more efficient in decreasing SDS symptoms than *S. tendae* UAE1 treatment. It is worth mentioning that the corresponding control seedlings, designated as *St* or *Sv*, looked good and had vigorous root systems.

It is essential to know how the modes of action and production of ACCD by BCAs affect their efficacy on conidial counts and DSI *in planta*. The numbers of conidia recovered in *St* + *Fs* and *Sv* + *Fs* seedlings were about three-fold less than of *Fs* (Figure 3C), suggesting that *S. tendae* UAE1 and *S. violaceoruber* UAE1 significantly (*P* < 0.05) reduced conidial counts to the same level. The DSI was also estimated on plants treated with *St* or *Sv* in soils infested with *Fs*. During the period of infection, SDS symptoms progressed on seedlings grown in soil colonized with *F. solani*, which was also reflected on the high DSI scores (Figure 3D). It was clear that the two BCAs had a substantial drop in DSI. Thus, the DSI estimates were greatly (*P* < 0.05) reduced in *Sv* + *Fs* seedlings when compared with that of *St* + *Fs* plants at 35 dpt.

To test if ACCD produced by *S. violaceoruber* UAE1 had additive protective effects on diseased seedlings, the ACC levels in the root and shoot tissues were quantitatively determined. Seedlings grown in soil with *St* only showed comparable amounts of ACC in their root and shoot tissues with those when no pathogen was applied (Figure 3E). However, seedlings grown in *Sv*-amended soil significantly (*P* < 0.05) reduced the ACC levels of the root and shoot tissues to the minimum compared to any other treatment. This suggests that seedlings can perform better when treated with the ACCD-producing *S. violaceoruber* UAE1.

Upon infection with *F. solani*, ACC levels significantly (*P* < 0.05) increased *in planta* compared to their corresponding control treatments (Figure 3E). Unlike *S. tendae* UAE1-treated soil, *S. violaceoruber* UAE1 decreased not only the disease severity (Figures 3A–D), but also ACC in diseased plants to almost the same levels as in non-diseased control plants (Figure 3E). The ACC levels were the same in the root and shoot tissues of *St* + *Fs* and *Fs* seedlings. Overall, our data indicate that the most effective BCA (i.e., *S. violaceoruber* UAE1) showing multiple antagonistic mechanisms and relatively high ACCD activities can suppress pathogen development and improve plant performance when exposed to fungal attacks.

### *In vitro* inhibitory effects of the culture filtrates of BCA1 and BCA2 on *F. solani*

**FIGURE 4:**
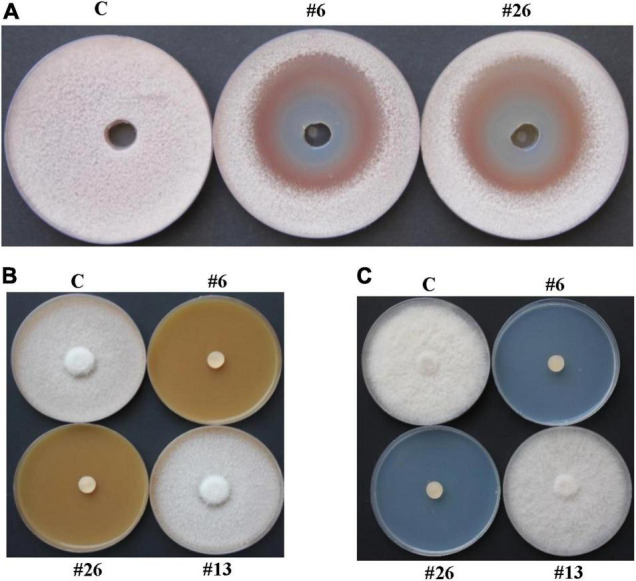
*In vitro* antifungal activities of BCA candidates against *Fusarium solani*. Inhibition of *F. solani* mycelial growth by actinobacterial isolates #6 and #26 using **(A)** cup plate method on PDA; and dialysis membrane overlay technique using **(B)** FMEA or **(C)** CCA plates. In **(A,B)**, inhibition of *F. solani* mycelial growth was observed by the diffusible antifungal metabolite-producing isolates #6 and #26. In **(C)**, inhibition of *F. solani* mycelial growth was observed by the chitinase-producing isolates #6 and #26. In **(A)**, wells are inoculated with either filter-sterilized FMEB **(C)**, or filter-sterilized crude culture filtrates of isolates #16 or #26; while in **(B,C)**, FMEA or CCA plates are either colonized by no BCA, or isolates #6, #13 or #26. BCA, biological control; PDA, potato dextrose agar; FMEA/B, fish meal extract agar/broth; CCA, colloidal chitin agar; C, sterile non-inoculated PDA agar plug/filter-sterilized FMEB (negative control); #13, *Streptomyces* sp. (positive control): #6, *Streptomyces tendae* UAE1 (ACCD-non-producing BCA1); #26, *Streptomyces violaceoruber* UAE1 (ACCD-producing BCA2).

Apart from the variation in ACCD production, the antagonistic activities of BCA1 and BCA2 were tested *in vitro*. When the culture filtrate of either BCA1 or BCA2 was applied using the cup plate method, there was a clear zone of inhibition in the growth of *F. solani*, compared to that when isolate #13 (positive control) was used (Figure 4A and Supplementary Table 2). This inhibition, however, was not significant (*P* < 0.05) on the plates containing the two BCAs and the pathogen, suggesting that the diffused antifungal metabolites of BCA1 and BCA2 were effectively performing almost the same on the growth of *F. solani*. In addition, the results obtained from the dialysis membrane overlay technique using FMEA plates indicated that the antifungal metabolites of BCA1 and BCA2 were fungicidal. This was evident when *F. solani* did not recover from the plugs transferred from BCA1- or BCA2-treated plates, but not isolate #13, to fresh PDA (Figure 4B).

In contrast to isolate #13, experiments obtained from the dialysis membranes using CCA plates of isolates (#6 and #26) showed that these BCAs completely inhibited *F. solani* growth *in vitro* (Figure 4C), suggesting fungicidal activities of their diffused CWDEs on *F. solani*. The enzymatic activities of chitinase and β-1,3-glucanase in both BCAs on media amended with colloidal chitin/*F. solani* cell walls and laminarin/*F. solani* cell walls, respectively, were also detected. No significant (*P* > 0.05) difference was found in the *in vitro* enzymatic activities between the ACCD-producing and ACCD-non-producing isolates, thus significant (*P* < 0.05) from isolate #13 (Supplementary Table 2).

In addition, the filter-sterilized crude culture filtrates of BCA1 and BCA2 were effective against *F. solani*. By increasing the culture filtrates of the BCAs to 50%, this significantly (*P* < 0.05) reduced the colony growth and mycelial biomass of *F. solani* after 5 days of incubation at 28°C on PDA plates, compared to no application of culture filtrates (Table 2). Thus, fungal growth, represented by colony diameter and mycelial dry weight, was completely retarded when 100% of the culture filtrate of BCA1 or BCA2 was applied. Although the reduction in conidial germination and germ tube elongation clearly occurred in *F. solani*, the fungal response did not differ due to the culture filtrates of BCA1 and BCA2 (Table 2).

**TABLE 2:** Effects of the crude culture filtrate of the BCAs on the morphological characteristic of mycelia, conidia, and germ tube of *Fusarium solani*.

Media	Isolate	Culture filtrate (%)	Colony diameter (mm)	Mycelial dry weight (g)	Conidia germination (%)	Germ tube length (μm)
FMEB	#6	0	97.6 ± 0.8 c	72.4 ± 1.0 c	82.4 ± 0.8 c	48.6 ± 2.0 c
		50	15.3 ± 1.2 b	8.1 ± 0.8 b	12.2 ± 1.9 b	10.7 ± 2.6 b
		100	0.0 a	0.0 a	1.1 ± 0.2 a	1.8 ± 0.3 a
	#26	0	97.2 ± 1.3 c	70.9 ± 1.4 c	80.6 ± 1.1 c	46.8 ± 1.5 c
		50	15.7 ± 1.0 b	8.8 ± 0.6 b	13.8 ± 1.5 b	9.9 ± 2.2 b
		100	0.0 a	0.0 a	0.9 ± 0.1 a	1.7 ± 0.4 a
CCB	#6	0	96.6 ± 0.4 c	69.4 ± 1.6 c	85.8 ± 0.8 c	62.3 ± 1.1 c
		50	13.5 ± 1.8 b	12.6 ± 1.0 b	16.7 ± 1.9 b	8.0 ± 0.8 b
		100	0.0 a	0.0 a	0.8 ± 0.1 a	0.0 a
	#26	0	95.8 ± 0.5 c	71.0 ± 1.2 c	84.4 ± 0.7 c	6163 ± 1.2 c
		50	13.6 ± 1.4 b	13.2 ± 0.9 b	15.8 ± 1.3 b	7.8 ± 0.7 b
		100	0.0 a	0.0 a	0.8 ± 0.0 a	0.0 a

Values are means ± SE of eight replicates. Values with the same letter within a column for each BCA are not significantly (P > 0.05) different according to Duncan’s multiple range test. Isolates #6 and #26 represent the ACCD-non-producing Streptomyces tendae UAE1 (BCA1) and ACCD-producing Streptomyces violaceoruber UAE1 (BCA2), respectively. BCA, biological control agent; FMEB, fish meal extract broth; CCB, colloidal chitin broth.

**FIGURE 5:**
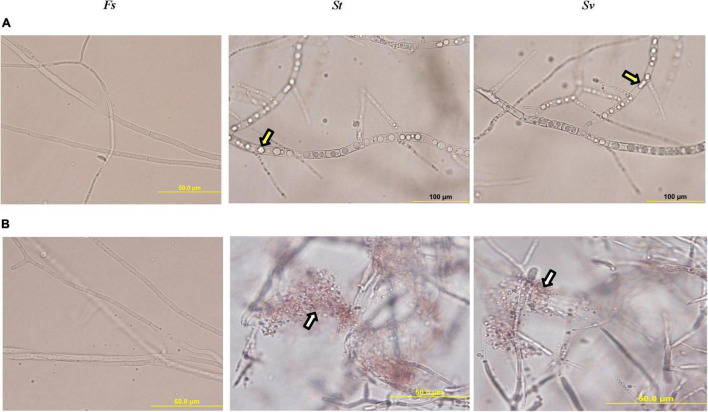
Inhibitory effect of the metabolites of BCA candidates on *Fusarium solani*. Abnormalities evident in the hyphal morphology and cytoplasmic contents of *F. solani*, following the treatment with filter-sterilized crude culture filtrate on **(A)** FMEB or **(B)** CCB of *St* (middle panel) and *Sv* (right panel) compared to control (left panel). Yellow arrows point to hyphal non-septate formation and cytoplasmic coagulation, while white arrows point to cytoplasmic lysis; respectively. *St*, the ACCD-non-producing *Streptomyces tendae* UAE1 (BCA1; isolates #6); *Sv*, the ACCD-producing *Streptomyces violaceoruber* UAE1 (BCA2; isolate #26); BCA, biological control agent; FMEB, fish meal extract broth; CCB, colloidal chitin broth. Light microscopy images were taken at 1,000 times total magnification.

When *F. solani* was treated with the filter-sterilized crude culture filtrate of any of the two BCAs, there were remarkable abnormalities in hyphal formation. This was clear from the aseptate (coenocytic) and branch-forming hyphae and coagulating cytoplasm of *F. solani*-treated with the culture filtrate of BCA1 or BCA2 on FMEB (Figure 5A). On CCB, lysis of fungal hyphae was observed in any of the BCA culture filtrate-amended flasks (Figure 5B). On the other hand, light microscopy detected healthy, unimpaired mycelial mats of *F. solani* in flasks that did not contain a BCA (control). Regardless of ACCD synthesis, *in vitro* BCA1 or BCA2 treatment had adverse effects on mycelial growth, spore germination, and germ tube elongation, ultimately causing membrane damage and outflow of cytoplasmic contents in *F. solani*.

## Discussion

Date palm diseases, such as SDS, associated with fungal pathogens are an emerging economical threat to the production of dates in the Arabian Peninsula, including the UAE. Microbial antagonists, also known as BCAs, for plant diseases prevent infection or establishment of the pathogen in host plants to control phytopathogens (Thambugala et al., 2020). Compared to other microorganisms, actinobacteria appear to withstand harsh conditions such as high temperature, salinity, and aridity (Dion, 2008; Qin et al., 2019; Xie and Pathom-aree, 2021). Therefore, we hypothesize that rhizospheric actinobacteria secreting ACCD along with a broad spectrum of antagonistic mechanisms against *F. solani* can substantially reduce the detrimental effect of SDS on date palm.

For that reason, several *in vitro* assays were carried out to give us a hint about the isolates showing multiple biocontrol properties to *F. solani* and possessing ACCD activities, even though this might not allow for drawing definite conclusions about them as effective BCAs *in vivo*. First, 54 actinobacteria, including 34 SA and 20 NSA, were isolated from rhizosphere soils of healthy date palm trees in the UAE. In general, *Streptomyces* species were found highly abundant, in alignment with other reports (Jose and Jebakumar, 2013; Palaniyandi et al., 2013). In addition, a series of *in vitro* assays were performed to determine the production of diffusible antifungal metabolites, CWDEs, VC, siderophores, and HCN by the isolated actinobacteria. According to Köhl et al. (2019), *in vitro* screening of microbial BCAs with multiple modes of action can potentially minimize the number of isolates to be used in the bioassay detection on plants or plant tissues. In the current study, ten (five from each SA and NSA) isolates with multiple modes of action were *in vitro* active against *F. solani* (Table 1), confirming the importance of actinobacteria as potential BCAs against plant pathogens (Saeed et al., 2017a; Kamil et al., 2018; Al Raish et al., 2021). It has been reported that inoculation of plants with ACCD-producing microorganisms can ameliorate the effect of environmental (biotic and abiotic) stresses in plants (Glick, 2014; Al Hamad et al., 2021; Glick and Nascimento, 2021; Jiménez-Mejía et al., 2022). In our study, the enzymatic activities of ACCD in these isolates were also measured. With four producing and six ACCD-non-producing isolates, preliminary studies for *in vivo* biological control under greenhouse conditions were evaluated.

According to their efficacy to SDS on date palm seedlings, two potential BCAs (isolates #6 and #26) were chosen. When tested for further biocontrol characteristics, both isolates confirmed their comparable production of diffusible antifungal metabolites and CWDEs (chitinase and β-1,3-glucanase) *in vitro*. Their culture extracts inhibited mycelial growth, conidial germination, and germ tube elongation of *F. solani* in a dose-dependent manner. Investigation of *F. solani* hyphae in the crude culture filtrate of BCA1 or BCA2 demonstrated that the hyphae showed abnormal growth and loss of internal content, resulting in a significant rise in the cellular leakage of *F. solani* mycelium. This suggests that the synthesis of secondary metabolites (i.e., diffusible and volatile antifungal substances) and the production of siderophores by the tested BCAs are involved in the inhibition of the growth of *F. solani*. Furthermore, the cell walls of *F. solani* which consist of chitin and glucan, are affected by chitinase and β-1,3-glucan enzymes produced by these BCAs. Except for ACCD production, almost similar *in vitro* biochemical results were obtained by *S. tendae* UAE1 (BCA1; isolate #6) and *S. violaceoruber* UAE1 (BCA2; isolate #26). Thus, the most effective BCAs studied to-date appear to antagonize pathogens using multiple mechanisms (Barka et al., 2016; Al Hamad et al., 2021; Alblooshi et al., 2022).

Genome sequencing of *S. tendae* UTMC 3329 has revealed important biological features of this actinobacterium tolerant to abiotic stresses (Eftekharivash and Hamedi, 2020), thus anticipating a future biotechnological and agricultural research. The siderophores-secreted *S. tendae* F4 has promoted the growth and improved the cadmium uptake in sunflower (Dimkpa et al., 2008). Activities of the chitinase and β-1,3-glucanase in ATCC 31160 and the expression of chitin-binding AFP1 protein from Tü901 have been attributed to the key role of nikkomycin-producing *S. tendae* as a bio-fungicide and bio-insecticide (Engel, 1987; Bormann et al., 1999). Thus, the current study reports the isolation and characterization of *S. tendae* UAE1 as a BCA to manage SDS on date palm in the UAE.

Researchers tend to apply a combination of isolates possessing multiple functions to maximize the effect to control phytopathogens. A mixture of actinobacterial, bacterial, or fungal isolates showing multiple modes of action has been reported effective in the suppression of diseases associated with soil-borne pathogens in plants (El-Tarabily et al., 2009; Thangavelu and Gopi, 2015a,b). In contrast, *S. tendae* UAE1 was used solely in the present study to enhance resistance in date palm against *F. solani*. Similarly, royal poinciana seedlings pre-inoculated with the endophytic *Streptomyces wuyuanensis* resulted in disease protection against *Neoscytalidium dimidiatum* (Al Hamad et al., 2021). This could be attributed to antibiosis and the production of CWDEs and other antagonistic substances (e.g., siderophores and/or VC) in *S. tendae* and *S. wuyuanensis*. Screening trials for a potential isolate with multiple mechanisms to serve as a BCA have identified *S. samsunensis* and *S. antibioticus* to manage dieback disease on mango and stem canker disease on royal poinciana, respectively (Kamil et al., 2018; Al Raish et al., 2021). Thus, these BCAs were more effective than other *Streptomyces* spp. possessing single modes of action.

Since *S. violaceoruber* UAE1 (BCA2) was relatively superior to *S. tendae* UAE1 (BCA1) as a BCA to manage SDS, we checked if the ACCD activity in the former *Streptomyces* species had additional effects on *F. solani*–date palm interaction (Figure 3). Previously, *S. violaceoruber* has been identified for its secondary metabolite products (Pham et al., 2005) and antibiotic properties (Duangmal et al., 2005). In addition, β-glucanase and phospholipases used as food additives (US FDA, 2007, 2015; Harazono et al., 2020), and cellulase and xylanase used in biological delignification (Abou-Dobara and Omar, 2014) are *S. violaceoruber* related-enzymes. This confirms the role of *S. violaceoruber* in many medical, industrial, and biotechnological applications (Duangmal et al., 2005). Yet, the inclusion of *S. violaceoruber* as a BCA in agricultural practices has not been reported.

Plant-emitted ethylene (ET), as a stress hormone, has received a considerable attention in the role it plays in resistance/tolerance to biotic/abiotic stresses at low concentrations. This can be achieved *via* the hydrolysis of ACC (the immediate precursor of ET) by the enzyme ACCD (Glick and Nascimento, 2021). It is widely reported that ACCD can degrade ACC to ammonia and α-ketobutyrate, thus reducing the level of ET *in planta* (Glick, 2014; Raghuwanshi and Prasad, 2018; Glick and Nascimento, 2021). Along with its biocontrol properties, *S. violaceoruber* UAE1 had additive effects of the suppression of SDS on seedlings in the greenhouse pot experiments. Similar to our results, Al Hamad et al. (2021) have demonstrated that the ACCD-producing *Streptomyces griseorubens* UAE2 had augmentative BCA effects on royal poinciana plants inoculated with *N. dimidiatum* compared to *S. wuyuanensis* UAE1 that did not have ACCD activity. This supports previously published studies reporting that plants inoculated with BCAs possessing ACCD activity can make the plant more resistant to environmental stresses (Wang et al., 2000; El-Tarabily et al., 2019, 2021; Mathew et al., 2020; Glick and Nascimento, 2021; Jiménez-Mejía et al., 2022).

Similar observations have been reported in non-actinobacterial isolates containing ACCD. Tomato plants treated with the ACCD-producing *Methylobacterium* spp. significantly reduced wilt symptoms of *Ralstonia solanacearum* and lowered the ET emission (Yim et al., 2013). Inhibition of crown gall tumors on tomato infected by pathogenic *Agrobacterium* strains was also associated with the application of ACCD-producing bacteria (Toklikishvili et al., 2010). In the current study, we claim that *S. violaceoruber* UAE1 is a more efficient BCA than *S. tendae* on date palm trees to control *F. solani*. This report extends current knowledge on an actinobacterial isolate producing ACCD and showing multiple antifungal characteristics to obtain *F. solani*-resistant date palm trees. This could potentially implement *S. violaceoruber* UAE1 in IDM programs. In addition to field studies evaluating *S. violaceoruber* UAE1 as a natural enemy to date palms infested with *F. solani*, omics-approaches (AbuQamar et al., 2016) predicting the outcomes of the molecular BCA-pathogen-plant interactions are on the top of our priorities.

## Data availability statement

The datasets presented in this study can be found in online repositories. The names of the repository/repositories and accession number(s) can be found in the article/Supplementary material.

## Author contributions

SFA and KE-T designed and conceived the research and supervised the study. KA, GP, ES, HA, SFA, and KE-T performed *in vitro* experiments. KA, GP, ES, SFA, and KE-T performed *in vivo* greenhouse experiments. GP and SFA developed the phylogenetic analysis. KA, GP, SFA, and KE-T analyzed the data. KA, HA, and SJA assisted with experiments and/or data evaluation. SFA and KE-T wrote the manuscript. All authors critically revised the manuscript and approved the final version.

## Footnotes

1
https://blast.ncbi.nlm.nih.gov/Blast.cgi

2
https://www.ncbi.nlm.nih.gov/nucleotide/
