# Correction: The 1-aminocyclopropane-1-carboxylic acid deaminase-producing *Streptomyces violaceoruber* UAE1 can provide protection from sudden decline syndrome on date palm

**DOI:** 10.3389/fpls.2026.1872068

**Published:** 2026-06-23

**Authors:** Khawla J. Alwahshi, Gouthaman P. Purayil, Esam Eldin Saeed, Haneen A. Abufarajallah, Shama J. Aldhaheri, Synan F. AbuQamar, Khaled A. El-Tarabily

**Affiliations:** 1 Department of Biology, College of Science, United Arab Emirates University, Al-Ain, United Arab Emirates; 2 Research Station Section, Abu Dhabi Agriculture and Food Safety Authority, Abu Dhabi, United Arab Emirates; 3 Khalifa Center for Genetic Engineering and Biotechnology, United Arab Emirates University, Al-Ain, United Arab Emirates; 4 Harry Butler Institute, Murdoch University, Murdoch, WA, Australia

**Keywords:** actinobacteria, biocontrol, date palm, *Fusarium solani*, rhizosphere, plant–microbe interaction, sudden decline syndrome

A correction has been made to the section **Materials and methods**, *In vivo greenhouse experiments*, Paragraph 7:

“All *in vivo* experiments were independently repeated three times with similar results.” has been corrected to “All *in vivo* experiments were conducted on three independent occasions to confirm reproducibility. Two independent greenhouse runs (8 plants per treatment per run) were pooled for statistical analysis (*n* = 16 plants per treatment) after confirming similar results across runs.”

A correction has been made to the section **Materials and methods**, *Statistical analysis*, Paragraph 2:

“Similar results were obtained in each replicate. For the fungal spore counts and DSI against *F. solani*, eight replicates for each experiment were examined. Data represent the mean ± SE from 16 plants. ANOVA and Duncan’s multiple range test were used to determine the statistical significance at *P* < 0.05.” has been corrected to “Each experiment included eight plants per treatment, resulting in a total of 16 plants per treatment. Data are presented as mean ± SE. Data were first analyzed using a one-way ANOVA, and treatment means were then compared using Duncan’s multiple range test at a significance level of *P* < 0.05. Mean values followed by the same letter are not significantly different, whereas those followed by different letters are significantly different from each other.”

A correction has been made to the section **Results**, *Identification of the promising BCAs*, Paragraph 3:

“BCA2 was assigned as *S. violaceoruber* (Waksman and Curtis, 1916) Pridham, 1970 strain UAE1.” has been corrected to “BCA2 was assigned as *S. violaceoruber* (Waksman and Curtis, 1916) Pridham, 1970 strain UAE1. It is worth mentioning that the suffix ‘UAE1’ for both *Streptomyces* species denotes the geographical origin of the isolates and is not intended as a unique strain identifier.”

A correction has been made to the section **Discussion**, Paragraph 4:

“Thus, the current study is the first to report the isolation and characterization of *S. tendae* UAE1 as a BCA to manage SDS on date palm in the UAE.” has been corrected to “Thus, the current study reports the isolation and characterization of *S. tendae* UAE1 as a BCA to manage SDS on date palm in the UAE.”

A correction has been made to the section **Discussion**, Paragraph 8:

The present study is the first report of an actinobacterial isolate producing ACCD and showing multiple antifungal characteristics to obtain *F. solani*-resistant date palm trees.” has been corrected to “This report extends current knowledge on an actinobacterial isolate producing ACCD and showing multiple antifungal characteristics to obtain *F. solani*-resistant date palm trees.”

The original version of this article has been updated.

