# Supplementary material for: The 1-aminocyclopropane-1-carboxylic acid deaminase-producing Streptomyces violaceoruber UAE1 can provide protection from sudden decline syndrome on date palm
Source: Front Plant Sci. 2022 Jul 27;13:904166. doi: 10.3389/fpls.2022.904166 (PMC9373858; doi:10.3389/fpls.2022.904166)
Supplement: Supplementary file 1 [file Data_Sheet_1.pdf]

Supplementary Material

The 1-aminocyclopropane-1-carboxylic acid deaminase-producing *Streptomyces violaceoruber* UAE1 can provide protection from sudden decline syndrome on date palm

Khawla J. Alwahshi, Gouthaman P. Purayil, Esam Eldin Saeed, Haneen A. Abufarajallah, Shama J. Aldhaheri, Synan F. AbuQamar* and Khaled A. El-Tarabily*

*** Correspondence:**
Synan AbuQamar: sabuqamar@uaeu.ac.ae
Khaled El-Tarabily: ktarabily@uaeu.ac.ae

Supplementary Figures

Supplementary Table S1. Comparison on the effect of application of polyvalent *Streptomyces* phages and dry heat techniques on the numbers of SA and NSA (cfu) isolated from rhizosphere soils of date palm trees.

Supplementary Figure S1. Colonies of actinobacteria isolated from date palm rhizosphere grown on arginine vitamin agar. Soil dilutions tubes were treated without (left) and with heat (right). White arrows represent the dominance of streptomycete actinobacterial colonies (left); whereas yellow and red arrows represent the dominance of non-streptomycete actinobacterial colonies *i.e., Actinoplanes* and *Micromonospora* spp., respectively (right).

Supplementary Figure S2. Antifungal activities and production of CWDEs by BCA candidates against *Fusarium solani*. *In vitro* screening for (A) inhibition of *F. solani* mycelial growth using the cut plug method; and production of (B) chitinase enzymes by BCA1 and BCA2 grown on colloidal chitin agar (upper panel) and production of CWDEs on mycelial fragment agar (lower panel). C, a sterile non-inoculated PDA agar plug (control). CWDEs, cell wall degrading enzymes; BCA, biological control agent; PDA, potato dextrose agar. Isolate #13 is a non-antifungal-, non-CWDE- and non-ACCD-producing positive control (*Streptomyces* sp.). Isolates #6 and #26 represents the ACCD-non-producing *Streptomyces tendae* UAE1 (BCA1) and ACCD-producing *Streptomyces violaceoruber* UAE1 (BCA2), respectively.

Supplementary Figure S3. Production of siderophores and ACCD by actinobacterial isolates. *In vitro* screening for production of (A) siderophores and (B) ACCD by selected actinobacterial isolates #6 and #26. In (A), isolates were tested on chrome azurol S agar plates; and yellow halo surrounding the colony of #6 and #26, but not #13 (positive control), indicated the excretion of siderophores. In (B), isolates were tested in N-free DF medium amended with ACC; where growth and sporulation in isolate #26, but not #6, indicated the efficiency to utilize ACC and production of ACCD. Isolate #13 is a non-antifungal-, non-CWDE- and non-ACCD-producing positive control (*Streptomyces* sp.); isolates #6 and #26 represents the ACCD-non-producing *Streptomyces tendae* UAE1 (BCA1) and ACCD-

producing *Streptomyces violaceoruber* UAE1 (BCA2), respectively; ACC, 1-aminocyclopropane-1-carboxylic acid; ACCD, ACC deaminase; DF, Dworkin and Foster's salts minimal agar.

Supplementary Figure S4. Taxonomic identification of the ACCD-non-producing *Streptomyces tendae* UAE1 (BCA1). (**A**) The dendrogram showing the phylogenetic relationships between *S. tendae* UAE1 (isolate #6; 1520 bp; OL356342) and other members of *Streptomyces* spp. on the basis of 16S rRNA sequences. (**B**) Light gray aerial (left) and yellow to greenish yellow substrate mycelia with the production of yellow pigment on the reverse side of culture (right) growing on ISP3 medium supplemented with yeast extract; and (**C**) scanning electron micrograph (6500X) of the spiral-shaped spores with 10-50 smooth-surfaced spores/chain of *S. tendae* UAE1 (spore chains belonged to section Spirales). In (**A**), numbers at nodes indicate percentage levels of bootstrap support based on a neighbor-joining analysis of 500 resampled datasets. GenBank accession numbers are given in parentheses. BCA, biological control agent.

Supplementary Table S2. *In vitro* comparisons of antagonistic activities between the two promising BCAs (*St* and *Sv*) against *F. solani*.

Supplementary Table S1. Comparison on the effect of application of polyvalent *Streptomyces* phages and dry heat techniques on the numbers of SA and NSA isolated from rhizosphere soils of date palm trees.

Actinobacteria	Without phage	With phage	Without heat	With heat
	log$_{10}$ cfu g soil^{-1}			
SA	8.20±0.76 *aA*	2.85±0.44 *bA*	7.90±0.52 *aA*	3.13±0.80 *bA*
NSA	2.16±0.44 *aB*	6.51±0.46 *bB*	1.75±0.66 *aB*	5.89±0.93 *bB*

Values are means±SE of eight replicates. For each method, values with the same upper or same lower case letter within a column or a row, respectively, are not significantly (P>0.05) different according to Duncan's multiple range test. SA, streptomycete actinobacteria; NSA, non-streptomycete actinobacteria; cfu, colony-forming units.

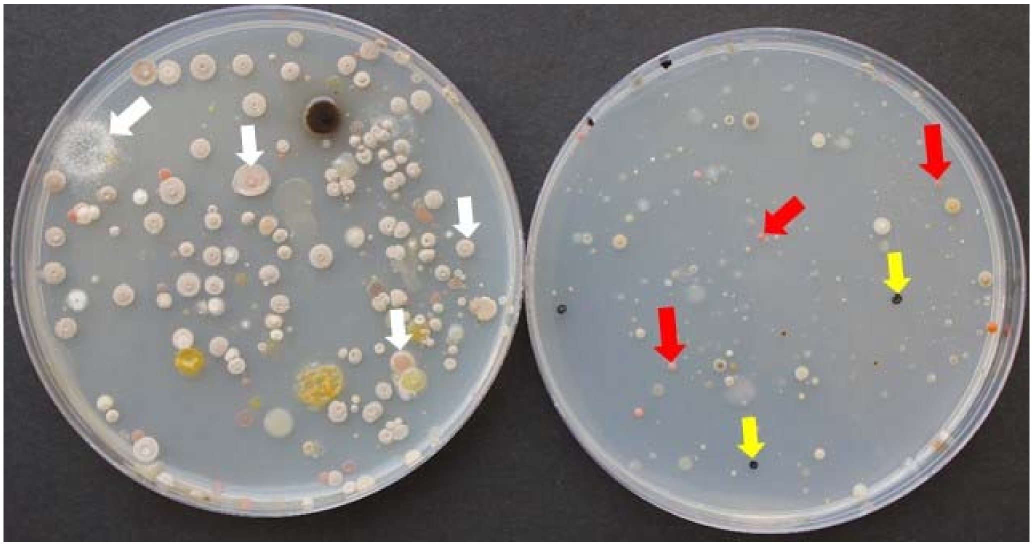

Supplementary Figure S1. Colonies of actinobacteria isolated from date palm rhizosphere grown on arginine vitamin agar. Soil dilutions tubes were treated without (left) and with heat (right). White arrows represent the dominance of streptomycete actinobacterial colonies (left); whereas yellow and red arrows represent the dominance of non-streptomycete actinobacterial colonies *i.e., Actinoplanes* and *Micromonospora* spp., respectively (right).

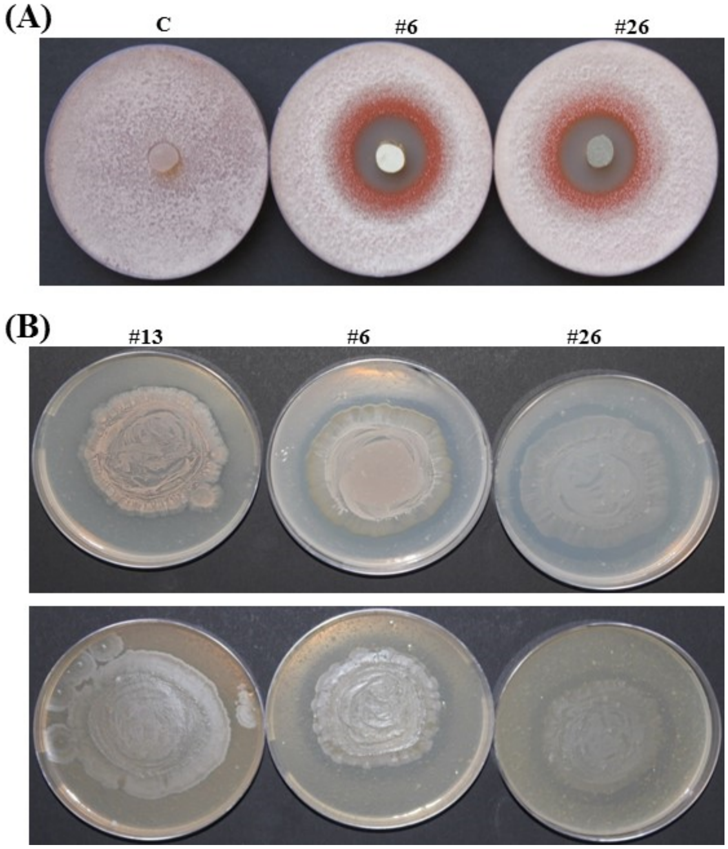

Supplementary Figure S2. Antifungal activities and production of CWDEs by BCA candidates against *Fusarium solani*. *In vitro* screening for (A) inhibition of *F. solani* mycelial growth using the cut plug method; and production of (B) chitinase enzymes by BCA1 and BCA2 grown on colloidal chitin agar (upper panel) and production of CWDEs on mycelial fragment agar (lower panel). C, a sterile non-inoculated PDA agar plug (control). CWDEs, cell wall degrading enzymes; BCA, biological control agent; PDA, potato dextrose agar. Isolate #13 is a non-antifungal-, non-CWDE- and non-ACCD-producing positive control (*Streptomyces* sp.). Isolates #6 and #26 represents the ACCD-non-producing *Streptomyces tendae* UAE1 (BCA1) and ACCD-producing *Streptomyces violaceoruber* UAE1 (BCA2), respectively.

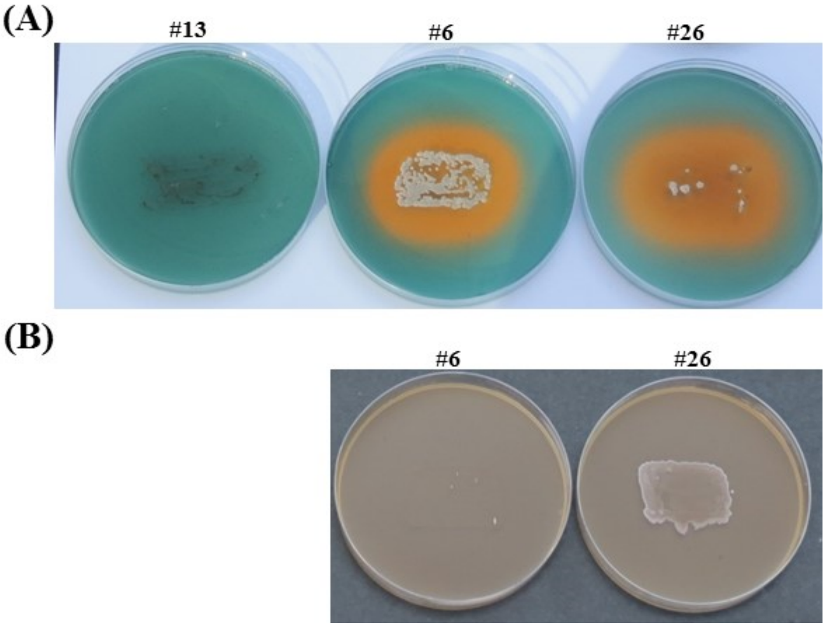

Supplementary Figure S3. Production of siderophores and ACCD by actinobacterial isolates. *In vitro* screening for production of (A) siderophores and (B) ACCD by selected actinobacterial isolates #6 and #26. In (A), isolates were tested on chrome azurol S agar plates; and yellow halo surrounding the colony of #6 and #26, but not #13 (positive control), indicated the excretion of siderophores. In (B), isolates were tested in N-free DF medium amended with ACC; where growth and sporulation in isolate #26, but not #6, indicated the efficiency to utilize ACC and production of ACCD. Isolate #13 is a non-antifungal-, non-CWDE- and non-ACCD-producing positive control (*Streptomyces* sp.); isolates #6 and #26 represents the ACCD-non-producing *Streptomyces tendae* UAE1 (BCA1) and ACCD-producing *Streptomyces violaceoruber* UAE1 (BCA2), respectively; ACC, 1-aminocyclopropane-1-carboxylic acid; ACCD, ACC deaminase; DF, Dworkin and Foster's salts minimal agar.

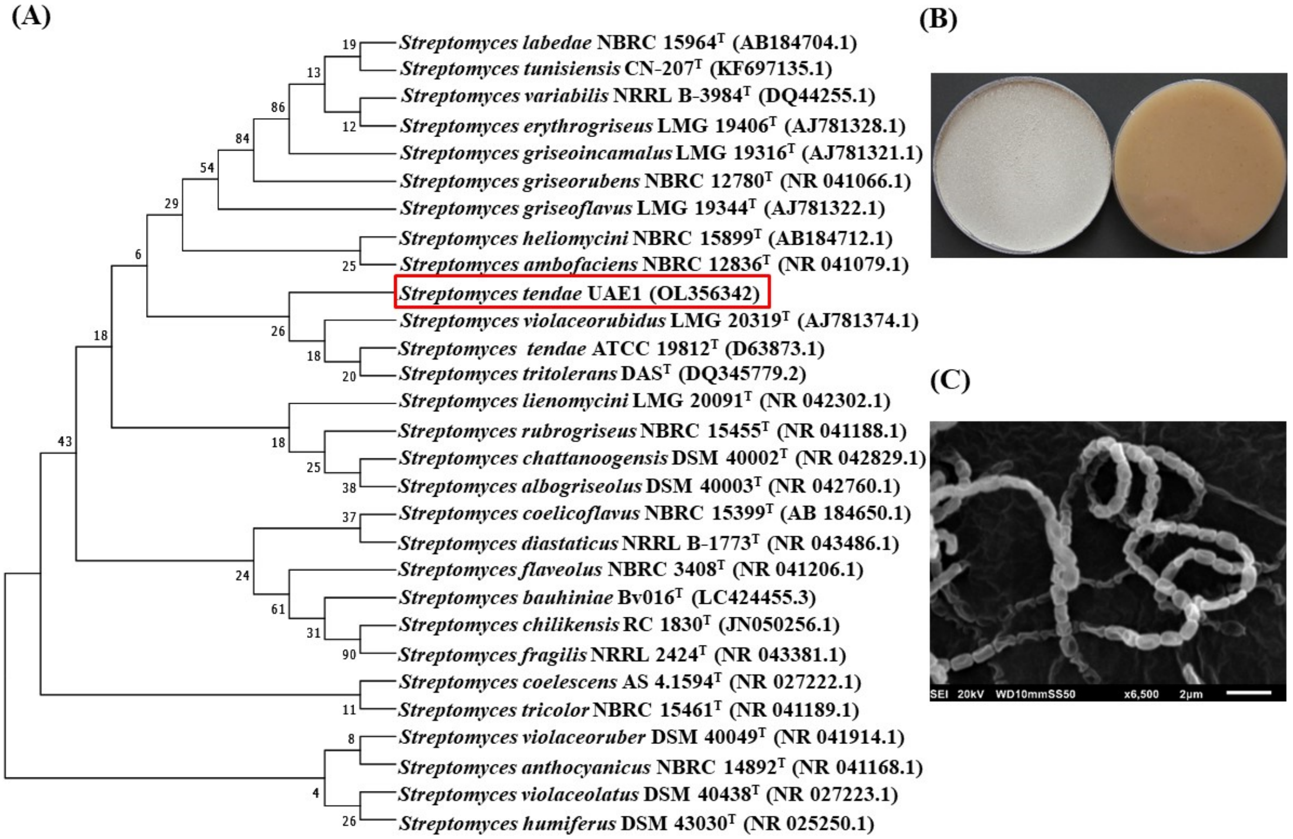

Supplementary Figure 4. Taxonomic identification of the ACCD-non-producing *Streptomyces tendae* UAE1 (BCA1). (**A**) The dendrogram showing the phylogenetic relationships between *S. tendae* UAE1 (isolate #6; 1520 bp; OL356342) and other members of *Streptomyces* spp. on the basis of 16S rRNA sequences. (**B**) Light gray aerial (left) and yellow to greenish yellow substrate mycelia with the production of yellow pigment on the reverse side of culture (right) growing on ISP3 medium supplemented with yeast extract; and (**C**) scanning electron micrograph (6500X) of the spiral-shaped spores with 10-50 smooth-surfaced spores/chain of *S. tendae* UAE1 (spore chains belonged to section Spirales). In (**A**), numbers at nodes indicate percentage levels of bootstrap support based on a neighbor-joining analysis of 500 resampled datasets. GenBank accession numbers are given in parentheses. BCA, biological control agent.

Supplementary Table S2. *In vitro* comparisons of antagonistic activities between the two promising BCAs (*St* and *Sv*) against *F. solani*.

**Activities**	**Isolate**		
	**#13**	***St***	***Sv***
**Production of diffusible metabolites using**			
cup plate (diameter of inhibition in mm)	2.3±0.4 *a*	59.7±2.3 *b*	60.2±2.4 *b*
**Chitinase from**			
colloidal chitin (U/mL) [a]	0.00 *a*	8.88±1.28 *b*	8.98±1.34 *b*
F. solani cell wall (U/mL) [a]	0.00 *a*	3.95±1.10 *b*	4.06±0.56 *b*
**ß-1,3-glucanase from**			
laminarin (U/mL) [b]	0.00 *a*	5.96±0.86 *b*	6.02±0.94 *b*
F. solani cell wall (U/mL) [b]	0.00 *a*	1.88±0.38 *b*	1.94±0.42 *b*

[a] One unit of chitinase activity: The amount of the enzyme required to release 1 µmol of N-acetyl-D-glucosamine/mg protein.h.
[b] One unit of ß-1,3-glucanase activity: The amount of the enzyme required to release 1 µmol of glucose/mg protein.h.
According to Duncan's multiple range test, values (means±SE) with the same row were not significantly (P>0.05) different from each other. Eight replicates/experiment were carried out with similar results.
St, the non-ACCD-producing *Streptomyces tendae* UAE1 (BCA1; isolate #6); *Sv*, the ACCD-producing *Streptomyces violaceoruber* UAE1 (BCA2; isolate #26). Isolate #13 is a non-antifungal-, non-CWDE- and non-ACCD-producing positive control (*Streptomyces* sp.). BCA, biological control agent.
